# Medication for opioid use disorder service delivery in carceral facilities: update and summary report

**DOI:** 10.1186/s40352-025-00317-9

**Published:** 2025-02-01

**Authors:** Justin Berk, Anna-Maria South, Megan Martin, Michael-Evans James, Cameron Miller, Lawrence Haber, Josiah Rich

**Affiliations:** 1 Alpert Medical School at Brown University, Providence, USA; 2 University of Kentucky, Division of Hospital Medicine and Addiction Consult and Education Service, Department of Medicine, University of Kentucky College of Medicine, Lexington, USA; 3 Denver Health and Hospital Authority, Division of Hospital Medicine, Department of Medicine, University of Colorado, Aurora, USA

## Abstract

The opioid overdose crisis intersects critically with the criminal legal system where individuals with opioid use disorder (are significantly overrepresented. Subsequently, incarceration increases the risk of opioid overdose due to reduced tolerance, interrupted social supports, and limited access to treatment. Medications for opioid use disorder (MOUD), such as methadone, buprenorphine, and naltrexone, have been shown to reduce opioid-related mortality and improve outcomes for those in carceral settings. Despite this, access to MOUD in jails and prisons remains limited due to stigma, concerns about medication diversion, and logistical challenges. This paper reviews the current state of knowledge on MOUD in carceral settings, summarizing the prevalence of treatment programs, the role of novel formulations like injectable buprenorphine, and barriers to implementation. It also explores the continuum of care, emphasizing the importance of initiating MOUD during incarceration and ensuring continuation upon release to prevent treatment gaps. Recent policy changes, such as Sect. 1115 Medicaid waivers, offer promising avenues for expanding access, but retention in treatment and post-release outcomes remain significant challenges. The paper provides a comprehensive overview of existing literature and updates on MOUD service delivery, including the impact of recent policy shifts and research on outcomes such as recidivism and health improvement. It concludes by identifying key areas for future research, including strategies to improve treatment retention, address systemic barriers through criminal justice reform, and enhance care coordination during the transition from incarceration to the community.

## The opioid overdose crisis and the criminal legal system

People with opioid use disorder (OUD) are overrepresented in the criminal legal system (CLS). Approximately one-third of people with OUD have encountered the CLS in the past year with greater than three quarters of individuals who use heroin reporting CLS involvement (Boutwell et al., 2007; Joudrey et al., 2019; Winkelman et al., 2018). Given this intersection between substance use and the criminal legal system, it is crucial to understand the heightened vulnerability to opioid overdose that incarceration imposes on individuals.

Incarceration increases the risk of opioid overdose and death (Binswanger et al., 2013; Farrell & Marsden, 2008; Merrall et al., 2010). This occurs through a number of mechanisms including a decrease in drug tolerance while incarcerated, the presence of comorbid medical conditions, exposure to socio-cultural norms or behaviors that encourage drug use upon release, and poor access to treatment facilities (Waddell et al., 2020). Upon release from prison, formerly incarcerated individuals then have a 129 times greater risk of opioid overdose death relative to the general population (Binswanger et al., 2007). One statewide study from Maryland suggested nearly half of fatal opioid overdoses occur in people with CLS involvement (Saloner et al., 2020).

Treatment of OUD during incarceration saves lives. When initiated in a carceral setting, medications for opioid use disorder (MOUD), including buprenorphine, methadone, and naltrexone, can significantly increase treatment engagement in the community upon release from a carceral facility and reduce opioid related mortality by over 50% (Green et al., 2018; Gordon et al., 2008; Kinlock et al., 2007; Marsden et al., 2017; Moore et al., 2019). The World Health Organization, National Institute on Drug Abuse, and the National Academy of Medicine endorse providing MOUD to incarcerated populations (National Academies of Sciences Engineering and Medicine, 2020; NIDA, 2014; World Health Organization, 2009).

Despite robust evidence on the effectiveness of MOUD and widespread organizational endorsement, few eligible individuals receive appropriate treatment while in jail or prison. The prevalence of OUD is high among incarcerated individuals, with approximately 1 in 5 individuals suffering from OUD (Joudrey et al., 2019). Yet studies suggest that among individuals meeting criteria for treatment, fewer than 10% ultimately receive MOUD during incarceration (Fox, 2015). Instead, even individuals receiving MOUD in the community often undergo forced opioid withdrawal upon incarceration, leading to decreased community treatment re-engagement and increased likelihood of overdose death (Rich et al., 2015). Potential barriers for MOUD uptake in jails and prisons include stigma, fear of diversion, resource needs for daily oversight of medication management, and cost (Brinkley-Rubinstein et al., 2019; Doernberg et al., 2019; Friedmann et al., 2012; Nunn et al., 2009). For those released without MOUD initiation, less than 5% ultimately receive this treatment in the community (Krawczyk et al., 2017).

A new policy landscape, however, continues to require that more carceral facilities offer MOUD to those detained. Federal court rulings affirm that it is illegal to withhold MOUD upon incarceration (Legal Action Center, 2024; Linden et al., 2018; Toyoshima et al., 2021). Withholding MOUD violates the American with Disabilities Act and can constitute cruel and unusual punishment under the Eighth Amendment of the U.S. Constitution (Legal Action Center, 2024; US Department of Justice, 2022). Legislation and state-level executive orders have mandated MOUD in several states (Carey & Longley, 2019; Toyoshima et al., 2021). Yet most carceral facilities still do not routinely offer all three forms of MOUD to incarcerated patients.

This paper describes the latest updates in addiction health services for OUD among incarcerated individuals including attempts to capture the prevalence of MOUD treatment programs in jails and prisons, the role for novel medication formulations (i.e., injectable buprenorphine), current data on diversion within facilities, effects of the Covid-19 pandemic on MOUD programs, updated post-release outcomes data, and discussion of facilitators and barriers to MOUD uptake in carceral facilities using an implementation science lens.

## An update on MOUD service delivery in carceral settings

### Medications for opioid use disorder

Medications approved for the treatment of opioid use disorder include methadone, an opioid agonist, buprenorphine, a partial opioid agonist, and naltrexone, an opioid antagonist. In addition to their ability to prevent opioid relapse and overdose, public and carceral health benefit of these treatments include a reduction in infectious disease transmission, including HIV and hepatitis (Mooney et al., 2020). As the three medications have a widely different mechanism of action, one cannot be simply replaced with a different one. The most common forms of MOUD found in carceral facilities are liquid methadone, sublingual buprenorphine, and injectable naltrexone.

Methadone is a schedule II medication under the US Drug Enforcement Administration (DEA). Within carceral settings, it is typically provided in liquid form to mitigate concern for concealing the medication in the mouth for later diversion (colloquially known as “cheeking”). To legally administer methadone for opioid treatment, carceral facilities must either go through the process of becoming a Substance Use and Mental Health Service Administration (SAMHSA) regulated Opioid Treatment Program (OTP) or partner with a community OTP (Rising et al., 2022). The formality required to administer methadone can serve as a barrier to providing the medication in carceral settings, especially for facilities that may not be in or near communities with a preestablished OTP (Rising et al., 2022).

Buprenorphine is similarly approved for the treatment of opioid withdrawal and maintenance therapy. It is typically administered sublingually through tablets or films, with some formulations also containing a fixed dose of naloxone as a misuse deterrent. Buprenorphine once required a special DEA “X” waiver to prescribe, though with recent federal policy changes, the medication can now be prescribed by any provider with an active DEA registration to prescribe Schedule III medications (Haber et al., 2023).

A major concern by correctional authorities for buprenorphine implementation in carceral facilities is diversion. Diversion of buprenorphine film or tablets does occur in carceral facilities, though recent qualitative studies suggest this happens to a limited degree and remains relatively preventable (Evans et al., 2022a; Evans et al., 2023). In fact, the implementation of MOUD programs can reduce the illicit buprenorphine market within carceral settings and the related coercion to divert their medication that people who are prescribed buprenorphine experience (Evans et al., 2022a).

Injectable buprenorphine has also been implemented in jails and prisons (Lee et al., 2021). This mode of administration may be preferable to some facilities and recipients, as it avoids medication diversion and can increase medical privacy by avoiding MOUD med-lines. Moreover, through longer administration intervals, the injection can provide several weeks of overdose protection at the time of community re-entry following jail or prison release- before individuals have engaged with an outpatient community provider at a crucial time when they are at the highest risk of returning to opioid use and overdose (Berk et al., 2022). Barriers to prescribing include injection site reactions and higher costs relative to sublingual buprenorphine formulations (Martin et al., 2022).

Naltrexone is a medication approved to treat both alcohol use disorder and OUD and may be prescribed by any licensed clinician. As the drug binds and blocks opioid receptors, there is little misuse or diversion potential within facilities. When compared to sublingual buprenorphine-naloxone, extended-release naltrexone can be more difficult to initiate patients on, resulting in an increased percentage of patients who decide against naltrexone initiation. Once initiated though both medications are equally safe and effective in terms of opioid relapse events, craving reduction, abstinent days, and overdose deaths (Lee et al., 2018). The strongest barrier to implementation is a commonly described treatment induction failure, followed by early return to use (naltrexone treatment retention is less than other opioid agonist treatments) (Lee et al., 2015). Ultimately, few individuals opt for this treatment choice while incarcerated (Clarke et al., 2018).

The goal of MOUD within carceral facilities is to offer those impacted by opioid use a safe, effective treatment option to mitigate the risk of withdrawal on carceral intake and to provide longitudinal management of a chronic disease both within the carceral facility and, ideally, on transition to the community at the time of release. Clinicians, health systems, and carceral systems should consider the variable formularies of jails and prisons in the region as well as available community resources. For example, initiating a patient on buprenorphine may be of more benefit while incarcerated if no methadone OTPs exist in the surrounding community for continuity of prescribing at release.

Of note, switching from one opioid agonist treatment to another (e.g., buprenorphine to methadone) during incarceration has been associated with decreased treatment retention after release, suggesting medication switching may be a possible way to identify more complex cases of OUD that would benefit from increased support during community reintegration (Carrieri et al., 2017; Curtis et al., 2022).

### Prevalence of MOUD programs

Public information on MOUD delivery across jails and prisons remains limited. Few facilities offer comprehensive treatment, including all three FDA-approved MOUD, other facilities offer only specific types of medication (e.g., injectable naltrexone) without patient-involved decision making, while some only offer treatment to specific populations (e.g., methadone in pregnant individuals) (Berk & Rich, 2021; Weizman et al., 2021). This variability between facilities can result in inadequate and inequitable medication access for incarcerated patients.

Several recent attempts have sought to characterize the prevalence of MOUD treatment in jails and prisons. The NIDA-funded Jail Prison and Opioid Project captures dynamic data on facility programs through web-scraping methodologies (Berk & Rich, 2021). The project found that less than half of all carceral facilities have public information about their MOUD programs and only 11.4% report access to all three forms of MOUD (Berk & Rich, 2021). In a recent 2022–2023 study querying a nationally representative cross-sectional survey of 1028 jails, less than half of jails (43.8%) offered medications for opioid use disorder to at least some individuals and 12.8% offered these medications to anyone with an opioid use disorder who requested them (Flanagan Balawajder et al., 2024). One recent study among jail facilities showed that 32% of jails reported MOUD availability in some capacity (Sufrin et al., 2023). This study also highlighted the limited information available: there was a 38% response rate, therefore opening the study to selection bias. The 2022 NIH Justice Community Opioid Innovation Network (JCOIN) National Survey of Substance Use Services in jails showed 43% of responding jails offered some type of MOUD (NORC at the University of Chicago, 2023).

As described above, uptake of potentially life-saving MOUD has been limited in prisons and jails. Current models predict that if all persons who were clinically indicated received MOUD while incarcerated and were retained in treatment post-release, 4400 deaths could be prevented annually (Macmadu et al., 2020).

### Facilitators and barriers to implementation of MOUD in carceral settings

Common barriers to implementation of MOUD in carceral settings described in academic literature include limited availability of addiction medicine trained providers within facilities, lack of availability of standardized treatment protocols, lack of funding from state, federal, or municipal budgets, or dedicated institutional space, stigma from correctional staff, institutional preference for abstinence-based treatment, and limited partnerships with community services (Ferguson et al., 2019; Grella et al., 2020). Similarly, the NIH JCOIN National Survey of Substance Use Services in Jails specifically asked about barriers to providing MOUD; respondents identified a lack of adequate licensed staff, policy prohibitions around MOUD, expense, and not enough individuals with OUD (NORC at the University of Chicago, 2023).

Facilitators to implementation include increased staff and staff training, group education, use of data-driven processes, coordination with other public safety agencies and community partners as well as ongoing contact with individuals post-release (Ferguson et al., 2019).

One recent scoping review of MOUD uptake in carceral facilities identified 4 categories of common barriers and facilitators: institutional, programmatic, attitudinal, and systemic. Institutional factors related to characteristics of the facility such as capacity, workforce, polices or regulations (Grella et al., 2020). Programmatic factors were defined as operations, practices, or interventions that are reflective within an institution. Attitudinal factors refer to attitudes, knowledge and beliefs among both participants of the programs and correctional staff. Systemic factors pertain to relationships or interactions between the criminal legal system and external service providers or service systems. For carceral settings with active MOUD programs or those seeking to initiate such, these categories serve as helpful framework for identifying and mitigating implementation barriers. Further, to help support facilities implementing new programs, an evidence-based checklist has helped stakeholders track benchmarks of success (Ludwig et al., 2022).

**Fig. 1:**
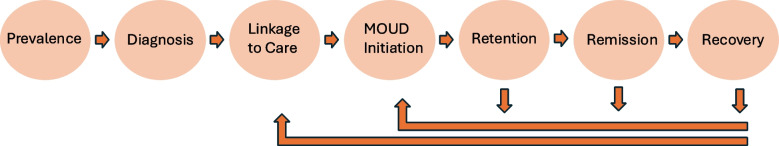
Cascade of care for treatment of opioid use disorder

A cascade of care model provides a valuable framework for identifying gaps in OUD and the pathway to recovery. The model seeks to monitor and improve care at various stages of health interventions and identify gaps in care by tracking drop-offs at different stages such as identification of need, referral, and treatment engagement. Several studies have tried to best characterize this pathway in substance use and often incorporate steps of prevalence, diagnosis, linkage to care, MOUD initiation, treatment retention, remission and recovery (Williams et al., 2022). Importantly, the chronic disease model of addiction highlights the relapsing nature of opioid use disorder, highlighting that individuals may have a non-linear path that includes return to use or return of OUD symptoms (see Fig. 1).

Incarceration or exposure to the criminal legal system and impact individuals at any step of the cascade. The cascade of care highlights the challenges associated with coordinating care across various agencies including the transition from carceral facilities to community-based treatment providers, where data demonstrates as a significant area for drop-out. (One study suggested found one in twenty adults referred for treatment within the criminal legal system ultimately received methadone or buprenorphine treatment) (Krawczyk et al., 2017).

Streamlining OUD identification and initiation among individuals in the criminal legal system requires multiagency collaboration, widespread education, and increased patient awareness of MOUD options (Clark et al., 2023). Beyond the barriers to implementation within carceral systems already discussed, further challenges arise during the transition from incarceration to the community—a high-risk period for disengagement from the cascade of care. These are discussed later in the paper.

## Special considerations

### Pregnancy

The American College of Obstetricians and Gynecologists (ACOG) and the Substance Abuse and Mental Health Services Administration (SAMHSA) recommend treatment with methadone or buprenorphine with individualized dosing for pregnant people with OUD, in conjunction with behavioral therapy and medical services, and continuation of MOUD after birth (Grella et al., 2020; Krawczyk et al., 2017; Ludwig et al., 2022; SAMSHA, 2018). Discontinuation of MOUD for OUD should be avoided during pregnancy as it can lead to preterm labor and loss of pregnancy, as well as in the time after delivery due to high risk of overdose with return to substance use. Currently naltrexone has limited evidence for use in pregnancy and is not recommended, though small sets of data have begun to suggest this could be a viable alternative for patients (Caritis & Venkataramanan, 2020). Methadone and buprenorphine are both safe and effective in pregnancy (Jones et al., 2012; Meyer et al., 2015).

Jails do not consistently provide pregnant people with access to medications that meet the standard of care for OUD treatment. In one national survey, only 60% of facilities reported continuation of MOUD for pregnant individuals who were receiving medication before incarceration, and only 32% of jails initiated MOUD during pregnancy (Sufrin et al., 2022). Similarly, gaps for MOUD treatment among pregnant patients exist in prison settings as well, with one analysis demonstrating only 40% of pregnant patients with OUD received MOUD during incarceration (Sufrin et al., 2022). Most patients were forced to discontinue MOUD in the postpartum period. This has significant health consequences given drug overdose has emerged as a leading cause of death among postpartum women (Frankeberger et al., 2023).

### Covid-19

During the Covid-19 pandemic, facilities had significant changes to their operations of MOUD delivery. To decrease person-to-person interactions, many facilities reduced their MOUD operations (Bandara et al., 2020). Among a multi-facility NIDA study, attempts to implement tele-health sought to sustain health services delivery in carceral settings. The majority of sites experienced challenges providing critical community support post-release, including referrals to housing, finding employment, and obtaining transportation services, all of which create challenges to treatment engagement and retention (Saunders et al., 2022).

### Outcomes

Implementation of MOUD has been associated with both health outcomes related to addiction and sequalae of injection drug use as well as other social outcomes within the criminal legal system (see Table 1). Table 1 provides information on prior studies conducted on outcomes of MOUD programs in carceral facilities, with statistical values (Odds Ratio and Hazard Ratio) for the outcomes included. The outcomes are separated into two categories: health outcomes and criminal justice outcomes.

**Table 1:** Selected outcomes associated with MOUD in carceral settings

OUTCOME	Treatment	Location/Setting	Statistical Value	Citation
**Health Outcomes**				
Reduced opioid use	Methadone		OR = 0.22 [0.15–0.32]	Moore et al. 2018*
	Naltrexone	Jail	OR = 0.08 [0.01–0.48]	Lee et al. 2015
	Naltrexone	Jail	OR = 3.5 [1.4–8.5]	Lee et al. 2015
Opioid Use	Methadone (vs. counseling)	Prison	OR = 4.68 [1.77–12.43]	Gordon et al., 2008
	Methadone (vs. counseling and transfer)	Prison	OR = 2.46 [0.95–6.37]	Gordon et al., 2008
	Naltrexone (1 month)	Jail	OR = 1.20 [0.48–2.94]	Farabee et al., 2020
	Naltrexone (3 months)	Jail	OR = 1.12 [0.37–2.81]	Farabee et al., 2020
	Naltrexone (6 months)	Jail	OR = 1.15 [0.33–3.93]	Farabee et al., 2020
	Naltrexone (12 months)	Jail	OR = 0.47 [0.16–1.35]	Farabee et al., 2020
Reduced injection use			OR = 0.26 [0.12–0.56]	Moore et al. 2018*
Community treatment retention	Methadone		OR = 8.69 [2.46–30.75]	Moore et al. 2018*
	Methadone	Prison	HR = 2.04 [1.48–2.80]	Rich et al., 2015
Fatal Overdose Risk	Methadone / Buprenorphine	Jail	aHR = 0.20 [0.08–0.46]	Lim et al., 2023
All-Cause Mortality	Methadone / Buprenorphine	Jail	aHR = 0.22 [0.11–0.42]	Lim et al., 2023
**Criminal Justice Outcomes**				
Arrest	Methadone	Jail	OR = 0.50 [0.35–0.72]	Evans et al., 2019
	Buprenorphine	Jail	OR = 0.49 [0.33–0.75]	Evans et al., 2019
	Naltrexone	Jail	OR = 1.44 [0.63–3.35]	Farabee et al., 2020
Re-incarceration / Recidivism	Methadone		OR = 0.93 [0.51–1.68]	Moore et al. 2018*
	Buprenorphine	Jail	HR = 0.71 [0.56–0.89]	Evans et al., 2022b
	Methadone	Jail	HR = 1.16 [0.8–1.68]	McMillan & Lackey 2008
Desistance			N/A	National Academy of Sciences

* Systematic review or meta-analysis

#### Health outcomes

MOUD are associated with lower rates of mortality, illicit opioid use, HIV transmission, criminal behavior, and recidivism in both the community and in carceral facilities (Connock et al., 2007; Cropsey et al., 2011; Dunlap & Cifu, 2016; Evans et al., 2019; Gisey et al., 2019; Gordon et al., 2008; Keen et al., 2000; Kinlock et al., 2009; Malta et al., 2019; Mattick et al., 2009; McKenzie et al., 2012; McMillan et al., 2008; Moore et al., 2019; Schuckit, 2016; Sordo et al., 2017; Wakeman et al., 2015; Wakeman et al., 2020).

Methadone and buprenorphine treatment for OUD during incarceration is associated with an 80% reduction in overdose mortality risk for the first month post-release (Lim et al., 2023). Studies consistently demonstrated improved community-based treatment engagement among individuals receiving methadone and buprenorphine while incarcerated (Gordon et al., 2008; Kinlock et al., 2007; Magura et al., 2009; McKenzie et al., 2012). Forced withdrawal of methadone treatment (i.e., discontinuing community MOUD on incarceration) decreases post-release treatment engagement (Rich et al., 2015). A 2019 meta-analysis showed that those receiving methadone during incarcerated had 8.69 times greater odds of community treatment engagement and a 78% reduction in illicit opioid use and injection drug use (Moore et al., 2019).

A 2023 systematic review of published peer-reviewed literature highlighted post-release outcomes associated with the use of MOUDs in carceral settings (Cates & Brown, 2023). Ultimately, this paper confirmed methadone and buprenorphine treatment during incarceration was associated with community-based treatment post-release and a significant reduction in post-release opioid use (though buprenorphine’s impact on opioid use post-release was more variable). The review highlighted mixed findings on recidivism across MOUD, with some evidence suggesting reduced criminal involvement among methadone and buprenorphine recipients.

#### Criminal legal system outcomes

Several studies have similarly demonstrated the positive effect of MOUD on post-release CLS involvement (e.g., re-arrest or re-incarceration) with methadone, buprenorphine, and methadone in women subpopulations. Similar findings have not been demonstrated with injectable naltrexone (Evans et al., 2022b; Farabee et al., 2020; Farrell-Macdonald et al., 2014; Gordon et al., 2008; Lee et al., 2015; Zaller et al., 2013).

While these are important outcomes to highlight, we should not use recidivism as the sole benchmark for public safety success. The National Academies of Medicine recently published guidelines on the limitations of recidivism as a marker for treatment success, instead calling for greater focus on “desistance,” or reduction in crimes committed, increase in time intervals between crimes, and decrease in severity of crimes (National Academies of Sciences E and M, 2022).

### Collateral benefits to carceral facilities

Notably, in-facility addiction treatment can provide collateral benefits to the carceral environment including improvements in public safety. Specifically, MOUD implementation may decrease contraband illicit substances, reduce staff burdens, and decrease violence (Brinkley-Rubinstein et al., 2019). These benefits can be particularly compelling for key decision-makers, especially security personnel, whose support is crucial for the success of MOUD programs but who may prioritize different goals than public health advocates.

### Policy and legislative considerations

The legal landscape supports a right to MOUD in carceral settings. In April of 2022, the Department of Justice released a statement to include OUD as a disability under the Americans with Disabilities Act (ADA) (US Department of Justice, 2022). A 2023 report from the O’Neill Institute provides a summary of federal efforts to ensure all federal prisons offer MOUD documents the growing momentum to expand access to MOUD in corrections nationwide. The documented growth in MOUD in all regions have been driven by a variety of factors including litigation, legislation, executive action, and guidance from the U.S. Department of Justice’s Civil Rights Division (Weizman et al., 2021). Other organizations such as the Legal Action Center provide a consistently updated review of case law related to MOUD access in jails and prison (Legal Action Center, 2024).

In one of the most recent legal ADA cases between the Department of Justice and one Kentucky jail facility, the final court ruling highlighted that it is illegal to force people to discontinue their MOUD upon incarceration, that people should be screened for OUD upon incarceration and be offered all three forms of MOUD (U.S. Attorney’s Office ED of K, 2023). A case from February 2024 showed this also applies to people who are involved with drug court or on parole; courts are not allowed to dictate people’s MOUD choices (Office of Public Affairs, 2024).

However, many facilities are not in compliance with the ADA and force patients to discontinue MOUD upon incarceration, highlighting the importance of continued clinician advocacy for increased access to MOUD (South et al., 2023). Courts have also cited the interpretation of the 8th Amendment of the US Constitution to allow continuation of MOUD after incarceration A recent commentary highlights how recent Supreme Court rulings may threaten this legal standard (Alsan et al., 2023).

In additional to court rulings and legislative mandates, an ethical argument for access to MOUD among incarcerated individuals has been present for decades (Bruce & Schleifer, 2008; Wakeman, 2017). When carceral policies prohibit MOUD, they prohibit a physician from carrying out the professional duty to do no harm (nonmaleficence). Withholding treatment may also violate principles of autonomy (if the patient desires such treatment), beneficence (as MOUD is evidence-based to reduce cravings and withdrawal), and justice (when treatment is readily available and accessible) (South et al., 2023).

### Financing, medicaid and costs

Unfunded policy mandates create an obvious challenge to MOUD implementation (Zhu et al., 2023). Costs associated with MOUD implementation fall to the municipalities that oversee jails and prisons. Federal policy mandates that Medicaid exclude coverage for individuals actively incarcerated. The Medicaid Inmate Exclusion Policy (MIEP) leaves the responsibility of healthcare funding to local, state, or federal carceral budgets. Of note, this interruption in coverage often results in worsened health outcomes and contributes to higher rates of recidivism as individuals face barriers to accessing necessary medical and mental health services upon reentry into the community (Albertson et al.,^ 2020^; Haber et al., 2024a, b).

Recently, policy shifts have aimed to address this gap in insurance coverage. The proposed federal Medicaid Reentry Act which aims to allow Medicaid coverage for certain services, including MOUD, during the 30 days prior to an individual’s release from incarceration. In 2023, CMS issued new guidance encouraging states to apply for a specific type of Sect. 1115 waiver, referred to as the Medicaid Reentry Sect. 1115 Demonstration Opportunity. This guidance allows states to cover select healthcare services, including MOUD for incarcerated individuals up to 90 days prior to their release (Centers for Medicare & Medicaid Services, 2023). As of August 2024, eleven states have approval to provide pre-release services to certain incarcerated, Medicaid eligible individuals (Hinton et al., 2024).

Limited data on the actual costs of MOUD programs are available. General community costs depend on multiple factors including frequency of clinic visits and counseling. A summary of medication costs in an OTP program for a stable patient estimates: Methadone, with daily support services, is $126 weekly ($6,552 annually). Buprenorphine, with bi-weekly support, is $115 weekly ($5,980 annually). Naltrexone, including all services, is $1,176.50 monthly ($14,112 annually) (NIDA, 2021).

In carceral settings, additional fixed costs may include accreditation fees, training, information technology services, planning meetings, and staff time associated with medication delivery (Ryan et al., 2023). A 2020 study in New Mexico conducted a cost-effectiveness analysis of a methadone maintenance treatment (MMT) program in a large urban jail, highlighting its impact on reducing incarceration days and associated costs. Individuals in the program exhibited almost 30 fewer days of incarceration due to decreases in recidivism over the subsequent year. The cost to reduce one incarceration-day using the MMT program was $23.49, far less than the cost of daily incarceration ($116.49) (Horn et al., 2020).

Injectable buprenorphine proves more cost-effective than tablets when considering the total costs of medication preparation, administration, monitoring, and personnel (Wong et al., 2022). A budget impact tool is available for facilities to estimate the resources and costs associated with alternative MOUD delivery models (Ryan et al., 2023).

### Barriers to treatment continuation and linkage to care

While Medicaid 1115 waivers can bridge some health insurance gaps at reentry, other insurance barriers significantly hinder treatment continuation post-release. For example, 1115 waivers will be a moot point for states that do not participate in Medicaid expansion as these individuals may stay uninsured after release from jail or prison. Moreover, access to MOUD varies significantly across state Medicaid programs. The SUPPORT Act of 2018 mandated that all Medicaid plans cover MOUD by January 2020 but did not eliminate utilization management policies like prior authorizations, which continue to obstruct access. Despite concerns that prior authorization policies create a barrier to MOUD access, these policies remain widespread in the nation’s Medicaid programs with one study showing approximately 50% of beneficiaries were subject to prior authorization for MOUD, with wide state variation (Abraham et al., 2022).

Research has identified additional barriers to treatment retention post-release. In one study, less than one-third of individuals receiving MOUD in jail continued care after release (Krawczyk et al., 2024). Qualitative research has highlighted barriers including structural factors including transportation, housing, basic resources, lack of employment, and fractured social networks stemming from incarceration (Hoffman et al., 2023; Kaplowitz et al., 2023). Resources including peer navigation supports have been identified by some as a facilitator for sustained treatment retention (Kaplowitz et al., 2023). Social supports and other wrap-around services including basic needs may be a critical support to ensure treatment engagement success upon release.

### Summary and potential policy

The opioid crisis remains a critical public health issue, with incarceration representing a high-risk predictor of mortality for people with OUD. Despite strong evidence supporting the effectiveness of MOUD in reducing post-release mortality, increasing treatment engagement, and reducing recidivism there remains a significant treatment gap within carceral settings. Public data on the prevalence of MOUD programs in carceral settings remains limited. Legal and ethical imperatives underscore the right to MOUD for incarcerated individuals, but policy and practice have been slow and incomplete in supporting MOUD uptake. The medical community can be proactive about addressing barriers to MOUD implementation in carceral settings and include new treatment approaches such as cost-effectiveness tools and different medication formulations (such as injectable buprenorphine).

Recent expansion of MOUD programs within jails and prisons, through both legislation and litigation, highlight the need for continued implementation research on the best manner in which to start and maintain these potentially life-saving programs. These advances within carceral facilities reveal a promising shift towards improved treatment and care continuity for incarcerated individuals with OUD, particularly during the high-risk time of community re-entry. Yet, despite medical evidence, legal advances, cost-effectiveness treatments, and a shifting policy landscape mandating treatment, the vast majority of facilities do not prescribe MOUD to patients that would benefit from treatment. This critical gap highlights a core public health priority affecting the two million individuals held in our jails and prisons and the nearly 10 million passing through each year.

### Looking forward

Key research gaps remain, particularly in implementing the evidence-based intervention of MOUD and care transitions into carceral facilities. A delay in adopting evidence-based interventions in carceral settings, also known as a “prison implementation penalty,” often delays the provision of gold standard care, including MOUD, in these settings (Berk et al., 2024). The growing field of implementation science is beginning to address these challenges, and, indeed, has begun to be applied to carceral facilities (Van Deinse et al., 2023; Zielinski et al., 2020). A National Institution of Health (NIH) funding mechanisms now exist to support implementation science into carceral healthcare research such as the National Institute of Drug Abuse’s Justice Community Opioid Innovation Network (JCOIN) which serves to study approaches to increase high-quality care for people in justice settings with opioid use disorder. Future health services delivery research should focus on gaps in implementing newer formulations of treatment (e.g., injectable buprenorphine); adapting screening tools for jail and prison settings; addressing stimulant use disorder in a carceral setting; understanding the effects of MOUD on institutional “climate,” violence, and disciplinary events; and streamlining data infrastructures for broader program evaluation. More broadly, reshaping correctional culture to focus on supporting rehabilitation rather than punishment, as observed in effective international carceral models, may have substantial public health benefits and enhance public safety outcomes (Ahalt et al., 2020).

Criminal justice reform has been identified as a crucial public health intervention, particularly in seeking to address health disparities (Haber et al., 2024a, b; Hon et al., 2024). Arguably, the most impactful way of addressing these health disparities would be to mitigate the epidemic of mass incarceration. as prison abolitionist Ruth Wilson Gilmore said “abolition is about presence, not absence. It’s about building life-affirming institutions.” Indeed, in 2020, the American Public Health Association published a policy statement supporting decarceration, which explicitly stated their recommendation of “moving toward the abolition of carceral systems and building in their stead just and equitable structures that advance the public’s health” (Conner et al., 2022). The criminal legal system has, in many cases, become a de facto safety net for individuals with addiction, severe mental health disorders, and even homelessness, often stepping in where community resources have failed to address these public health issues. However, relying on incarceration to provide essential services is neither just nor humane. Future efforts must focus on building life-affirming institutions within communities that can truly support individuals suffering from opioid use disorder and improve health outcomes for all, especially those currently incarcerated.

## Data Availability

No datasets were generated or analysed during the current study.
